# Molecular dissection of CRC primary tumors and their matched liver metastases reveals critical role of immune microenvironment, EMT and angiogenesis in cancer metastasis

**DOI:** 10.1038/s41598-020-67842-5

**Published:** 2020-07-01

**Authors:** Jiangang Liu, Yong Beom Cho, Hye Kyung Hong, Song Wu, Philip J. Ebert, Steven M. Bray, Swee Seong Wong, Jason C. Ting, John N. Calley, Catherine F. Whittington, Shripad V. Bhagwat, Christoph Reinhard, Robert Wild, Do-Hyun Nam, Amit Aggarwal, Woo Yong Lee, Sheng-Bin Peng

**Affiliations:** 1 Eli Lilly and Company, Lilly Corporate Center, Indianapolis, IN 46285 USA; 2 Department of Surgery, Samsung Medical Center, Sungkyunkwan University School of Medicine, 81 Irwon-Ro, Gangnam-Gu, Seoul, Republic of Korea; 3 Department of Health Science and Technology, Samsung Advanced Institute for Health Science and Technology, Sungkyunkwan University, Seoul, Republic of Korea; 4 Institute for Future Medicine, Samsung Medical Center, Seoul, Republic of Korea; 5 Department of Neurosurgery, Samsung Medical Center, Sungkyunkwan University School of Medicine, Seoul, Republic of Korea

**Keywords:** Cancer, Computational biology and bioinformatics, Molecular biology

## Abstract

Metastasis is the primary cause of cancer mortality. The primary tumors of colorectal cancer (CRC) often metastasize to the liver. In this study, we have collected 122 samples from 45 CRC patients. Among them, 32 patients have primary tumors, adjacent normal tissues, and matched liver metastases. Thirteen patients have primary tumors without distant metastasis and matched normal tissues. Characterization of these samples was conducted by whole-exome and RNA sequencing and SNP6.0 analysis. Our results revealed no significant difference in genetic alterations including common oncogenic mutations, whole genome mutations and copy number variations between primary and metastatic tumors. We then assembled gene co-expression networks and identified metastasis-correlated gene networks of immune-suppression, epithelial–mesenchymal transition (EMT) and angiogenesis as the key events and potentially synergistic drivers associated with CRC metastasis. Further independent cohort validation using published datasets has verified that these specific gene networks are up regulated throughout the tumor progression. The gene networks of EMT, angiogenesis, immune-suppression and T cell exhaustion are closely correlated with the poor patient outcome and intrinsic anti-PD-1 resistance. These results offer insights of combinational strategy for the treatment of metastatic CRC.

## Introduction

CRC is the third most common cancer in world with second highest cancer-related mortality worldwide^1^. In US alone, it is estimated that approximately 137,000 people are diagnosed, and more than 50,000 are dead from CRC each year. CRC primary tumors often metastasize to the liver, which accounts for most of CRC related death. The molecular mechanism of tumor metastasis remains poorly understood. It is believed to be a multiple step process that includes cells to detach from their original site and invade the neighboring submucosa, extravasate and survive in the vasculature and metastatic site, and eventually reestablish tumor in alien organ^2^. Prevention of tumor metastasis is dependent upon the better understanding of the molecular mechanism governing this complicate process. However, the extensive interactions among tumor cells and tumor microenvironment (TME) have complicated the efforts in dissecting the metastatic process^3^. There is no convincing evidence to date suggesting that the metastatic process links to specific genetic alterations in CRC^4^.

Most of CRCs are epithelial in origin, and the TME composition changes as the tumor grows and spreads^5^. The TME consists of extracellular matrix (ECM), cancer-associated fibroblasts (CAFs), endothelial cells, immune cells, and many soluble factors required for cancer progression^6^. The interaction between tumor and adjoining stromal tissues is an important aspect of the tumorigenic process and drug response^7,8^. For example, it has been reported that epithelial CRC cells could induce changes of normal fibroblasts into CAFs via secretion of transforming growth factor β (TGFβ)^9,10^. At the same time, CAFs may secrete growth factors such as fibroblast growth factor (FGF), platelet-derived growth factor (PDGF), and vascular endothelial growth factor (VEGF) to promote cancer cell proliferation and invasion^11,12^. Studies have also suggested that the stromal compartment plays an important role during cancer development and metastasis^13^. Besides TME-tumor interactions, EMT is a crucial process for metastatic cascade in which cancer cells transition from an epithelial cell type into a more invasive mesenchymal cell type for dissemination^14^. Recent studies have revealed that tumor cells and immune cells can reciprocally influence each, suggesting a potential role of immune microenvironment in EMT and tumor metastasis^14,15^. However, the understanding of each TME components contributing to the tumor metastasis and the dynamic cellular process remains elusive.

It has been a significant challenge for computational biology to deconvolute the genome-wide molecular networks. However, with recent advance in bioinformatic analyses, a few studies have demonstrated the feasibility to dissect the transcriptional networks from gene expression profiles^16^. Several methods are reported for such analyses, and one of them is called weighted gene co-expression network analysis^17^ (WGCNA). In the present study, we genomically characterized 109 samples from 45 human CRC patients, including primary tumors, their matched adjacent normal tissues and liver metastatic biopsies from 32 patients. We utilize WGCNA and cell-type deconvolution approaches to perform a virtual dissection of primary and metastatic CRC samples to allow us to identify tumor-specific, stromal cell-specific, and metastatic program-specific molecular modules with prognostic and biological relevance. We reveal molecular interactions among EMT, angiogenesis, and immunosuppression, three key drivers of cancer progression and their possible link to CRC metastasis.

## Results

### Patient and sample information

Our patient cohort consisted of 45 CRC patients with 32 patients having “trios” of primary CRC tumors (CWM, n = 32), adjacent normal samples (AN, n = 32) and patient-matched liver metastases (CLM, n = 32). Thirteen patients with primary tumors lacking any distant metastasis (CNM, n = 13) were included as baseline for comparison. The histology of all available samples was reviewed by a single pathologist blinded to sample identity. The clinical characteristics, patient follow up information and Microsatellite Instability (MSI) status of the patients in our cohort were summarized in Supplementary Table S1. We analyzed these samples using three genomics platforms: whole-exome sequencing for somatic mutations, array-based methods for profiling somatic copy-number changes, and RNA sequencing for mRNA expression (Supplementary Figure 1).

### A high degree of similarity in genomic alterations in CRC patients with and without distant metastases, and in primary tumors and the matched liver metastases

**Figure 1:**
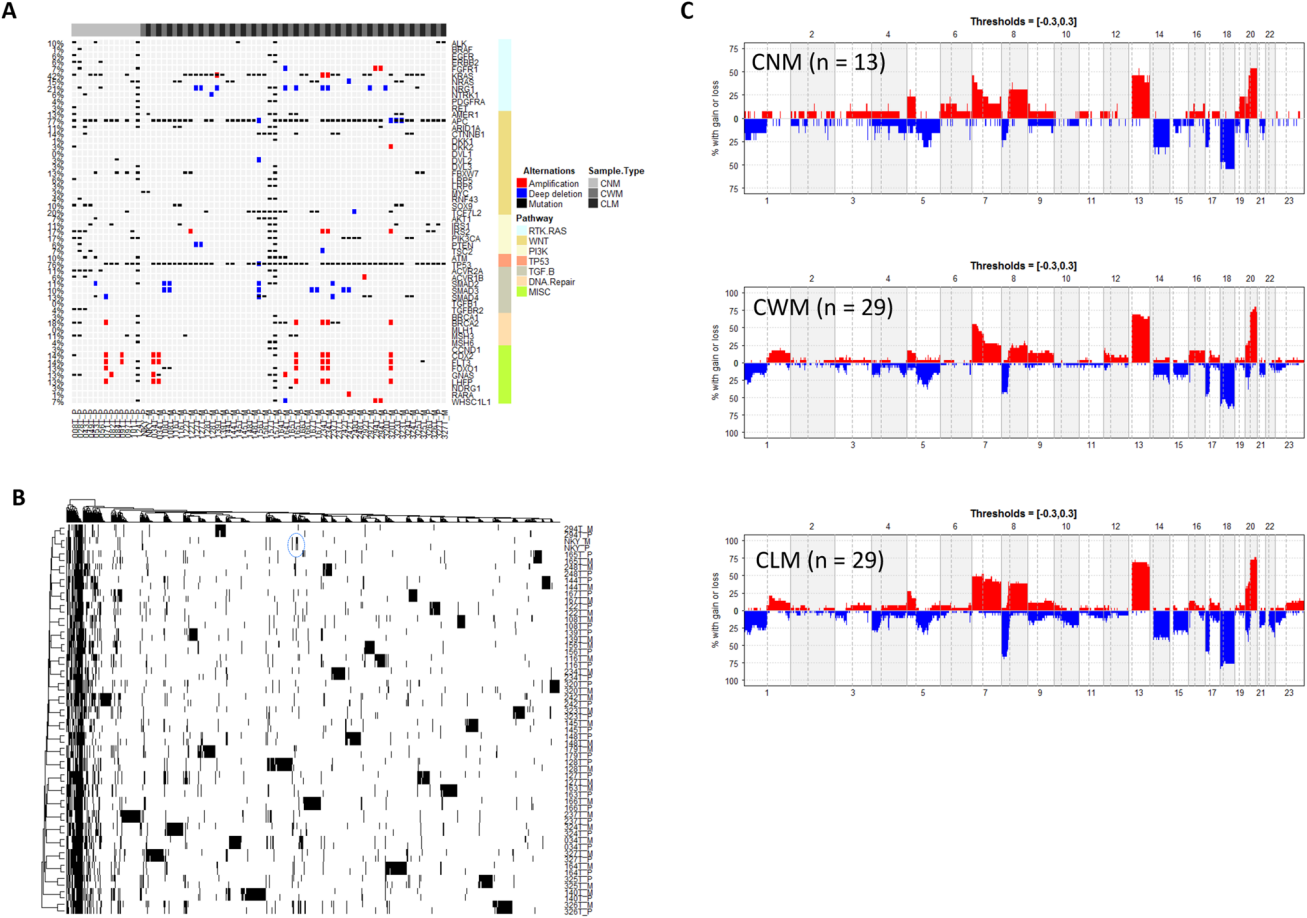
Comparison of the genetic aberrations in the primary tumor and matched liver metastases. (**A**). Somatic variants identified in genes grouped by deregulated signaling pathways in CRC. Mutation—non-synonymous single nucleotide or small indel mutation, Amplification—copy number amplification (CN >  = 4). Deep deletion—copy number deep deletion (CN <  = − 2). CNM—CRC with No Metastasis, CWM—CRC With Metastasis, and CLM—CRC Liver Metastases. The heatmap was generated using ComplexHeatmap^58^ (R package, version 1.18.1). (**B**) Hierarchical clustering of whole genomic alterations, made with gplots (R package, version 3.0.1.1; https://cran.r-project.org/web/packages/gplots/index.html), shows tightly aggregated primary tumor and matched metastasis regardless of clinical and/or pathological parameters. The mutations for cancer driver genes such as APC, TP53, KRAS etc. were clustered together that shared in most of primary and metastatic tumors, while the private mutations formed the patient-specific clusters. (**C**) No major difference was found in the frequencies of significant aberration in SCNAs of gain and loss areas between primary CRC and matched liver metastases in CRC patients. The plot was generated using Copynumber^59^ (R package, version 1.24.0).

To search for potential metastasis-related genetic alterations, we first investigated genetic mutations leading to deregulation of signaling pathways in CRC^18^. These pathway genes showed a similar profile and frequency of mutations in these samples (Fig. 1A) except for four hypermutated samples. These four hypermutated samples were all in CNM group, and three of them were MSI using immunohistochemical (IHC) staining of tumor tissues to detect loss or down-regulation of mismatch repair genes (including MSH2, MSH6, and MLH1; Supplementary Table S1). We noticed no significant difference in frequency of alteration in CRC associated genes in paired CWM samples versus the matched liver metastases (CLM) (Supplementary Table S2a & S2b). Similarly, we found no significant difference in the frequency of gene mutations between CWM and CNM if the four hypermutated samples are excluded from the comparison (Supplementary Table S2a & S2b). When we included the four hypermutated samples in analysis, only the prevalence of gene BCL6 corepressor (BCOR) mutation was significantly higher in CNM group (Fisher test, *p* = 0.007). The most frequently mutated cancer genes were *APC *(77%), *TP53* (76%), *KRAS* (42%), *NRAS* (15%), and *PIK3CA* (17%), in this cohort. Notably, mutations in *APC, TP53*,* KRAS*,* NRAS* and *PIK3CA* were more than 90% concordant between primary tumors and metastases (Supplementary Table S2c). Overall, we noted that the overall pattern of mutations detected in CNM, CWM and CLM patients was also highly similar (Supplementary Table S2a & S2b), confirming the results observed in previous reports^4,14,19^. We then extended the scope of mutation interrogation from these CRC-specific pathway genes to the whole-exome between 30 primary tumors (CWM) and their matched metastasis in livers (CLM) (2 pairs of samples were excluded after quality assurance). Hierarchical clustering analysis tightly aggregated primary tumor and matched metastasis together (Fig. 1B). The mutations from liver metastasis biopsies were highly similar to its matched primary tumors, but were divergent from each other among patients, suggesting that there was no significant difference between primary and metastatic tumors.

We next analyzed the somatic copy number alteration (SCNA) by array-based comparative genomic hybridization (aCGH) in CNM, CWM and CLM samples. We estimated the frequency of the gain or loss of each gene to calculate an amplification (red, CN > 4.0) or deletion (blue, CN < 1.0) score in each sample as shown in Fig. 1C. The comparison of CWM versus CNM as well as CLM showed that they exhibited no significant differences in their SCNA profiles.

Overall, our results demonstrated a high degree of similarity in genomic alterations in CRC patients with and without distant metastases, and in primary tumors and the paired metastatic biopsies, which is suggestive of relatively stable clonal evolution after tumor metastasis.

### Transcriptional differences between primary and metastatic tumors or primary tumors without and with metastasis

**Figure 2:**
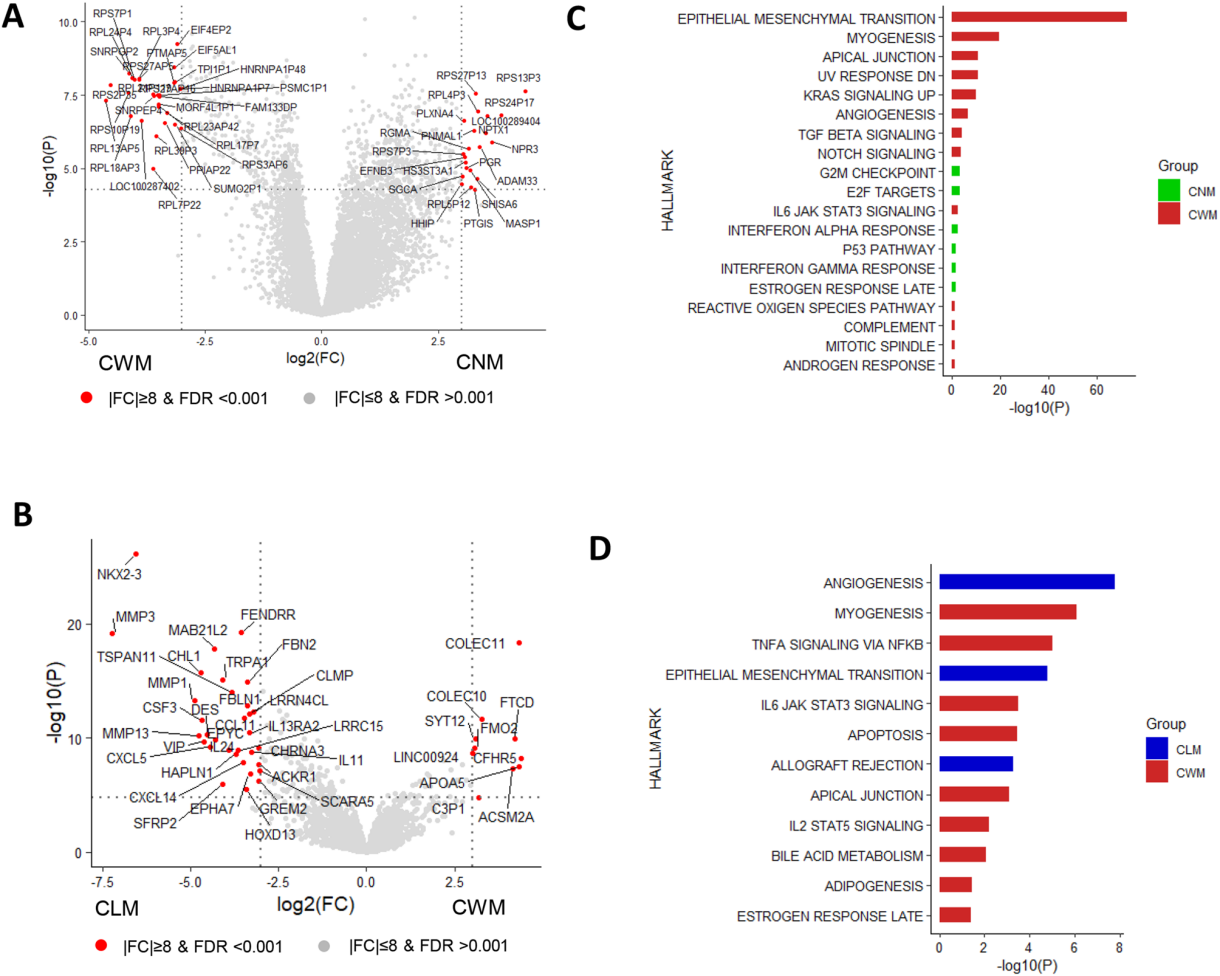
Transcriptional differences between the primary tumor and matched liver metastases. (**A**&**B**) Volcano plots for CWM versus CNM (**A**) and CLM versus CWM (**B**) were made using ggplot2 (R package, version 3.2.1; https://cran.r-project.org/web/packages/ggplot2/index.html). The distribution of the gene expression log2 fold changes (FC) versus -log10 p-values are shown. Genes with absolute fold change ≥ 8 and False Discovery Rate (FDR) < 0.001 are labeled with gene symbol and indicated in red color. P and FDR values are generated using R package “DESeq2”^20^ with Benjamini–Hochberg testing correction. (**C**&**D**) Summary plots for the over representation analysis (ORA) of cancer hallmark pathways^21^ for CWM versus CNM (**C**) and CLM versus CWM (**D**). The enrichment score (− log10 (*p* value)) is calculated for the Differential Expressed Genes (DEGs) present in each pathway using hypergeometric test. DEGs are identified with absolute fold change ≥ 2 and FDR < 0.05 between log2 normalized expression in the comparison of CWM versus CNM (**C**) or CLM versus CWM (**D**). Only the pathways with FDR ≤ 0.05 are displayed in the plot.

Given the absence of any significant genetic changes, we then compared the transcriptomic profiles between CWM and CNM, or between CWM and CLM by analyzing the RNA Seq data from 31 CLM-CWM paired samples (1 pair of samples did not pass the quality control) and 13 CNM samples using DESeq2^20^. The distributions of the fold changes and p-values of genes in each group were shown in Fig. 2A, B as volcano plots. We identified 520 up-regulated and 133 down-regulated Differentially Expressed Genes (DEGs) in the CWM versus CNM with absolute fold change ≥ 2 and FDR ≤ 0.05. Using the same criteria, 16 upregulated and 70 down-regulated genes in CLM group were identified from the pairwise comparison of CLM versus CWM (Supplementary Table S3). Functional analysis of the DEGs with 50 MsigDB cancer hallmark gene sets^21^ revealed distinct functional differences among groups. The gene set involving EMT and myogenesis was the most significantly upregulated pathway in CWM compared to CNM (Fig. 2C). Moreover, angiogenesis and inflammatory response were markedly enriched in CLM compared to CWM (Fig. 2D).

### Deconvolution of the transcriptional network shows TME-enriched modules are strongly correlated to metastasis

Our transcriptomic comparison detected 653 differentially expressed genes from CWM versus CNM and 86 genes from CLM versus CWM. However, the magnitude of expression differences from CLM-CWM pairwise comparison was small, posing a challenge to inferring their biological differences. We then focused our analysis on searching potential candidate genes or pathways that may underlie the metastatic process based on co-expression transcriptional network. Unlike the conventional single gene differential expression analysis, we used the weighted gene co-expression network analysis (WGCNA) to examine gene-to-gene relationships and to identify modules of coordinately expressed genes in an unsupervised way^17^ (Supplementary Figure S2). WGCNA used correlations to group genes into modules, and raised each correlation to a power, thus lending more weight to stronger, more reliable correlations. It then correlated these modules with binary vectors to clinical traits such as sample groups (CNM, CWM, and CLM) or metastatic status (yes or no). For example, a sample was assigned as 1 if it was metastatic or 0 if not. Therefore, a significant module-group correlation implies that samples from one specific group have higher expression than those of other groups.

**Figure 3:**
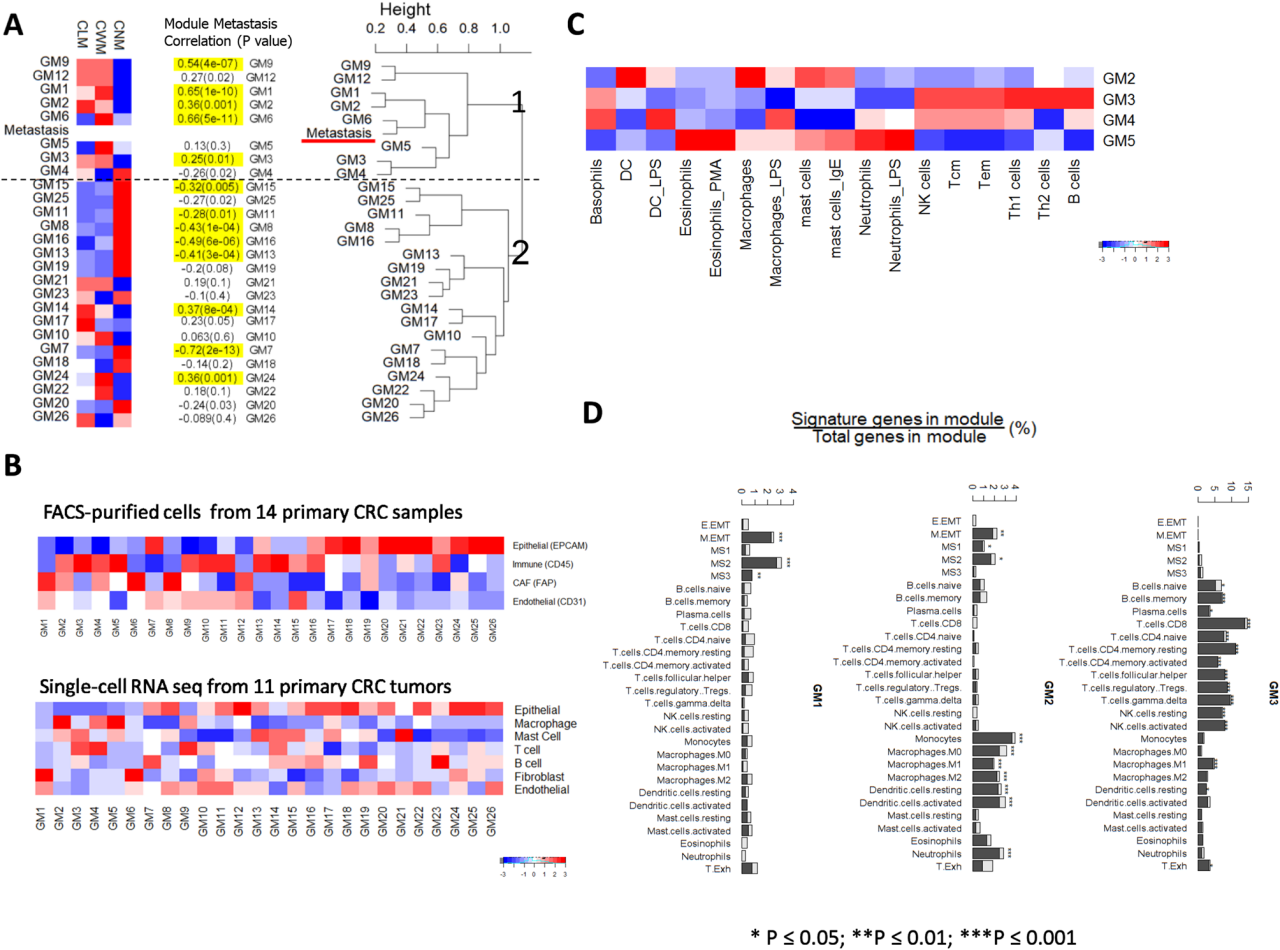
Deconvolution of 26 transcriptional modules and its correlation with clinical phenotype—Metastasis. (**A**) Left panel: heatmap shows the relative expression levels (eigengene expression) of each module; Middle panel: The module—phenotype “Metastasis” correlation coefficients with p values; The correlation with a *p* value ≤ 0.01 is highlighted with yellow color. Right panel: The hierarchical clustering dendrogram of module eigengenes and the phenotype “Metastasis”. GM 1—6, GM9 and GM12 are closely clustered with “Metastasis” status in branch 1. (**B**) Heatmap shows relative expression levels of each module in the FACS-purified cells from 14 primary CRC samples (Top panel; data from GSE39396) and the single-cell RNA seq from 11 primary CRC tumors (Bottom panel; data from GSE81861). (**C**) Deconvolution of immunity-related modules shows immune cell specific expression patterns: transcriptome data for purified adaptive and innate immune cells (immunome) were downloaded from GEO (GSE3982). (**D**) Overlap of the previously described EMT (E.EMT and M.EMT) and angiogenesis (MS1-MS3), T cell exhaustion (T.Exh), and Immune cell—specific signature with co-expression modules (the solid part of a given bar), along with the distribution of a random signature of equivalent size (the hollow part of a given bar) (overlap = signature genes/module genes, as a percentage). GM1-GM3 are used here as examples. The *p* values are generated with Chi-Square Test. The heatmaps were plotted using R package gplots (version 3.0.1.1; https://cran.r-project.org/web/packages/gplots/index.html ).

WGCNA analysis identified 26 modules of co-expressed genes, and the expression of representative genes in each module was mathematically summarized as an eigengene value^17^. The eigengene value of 26 modules in each group is displayed as a heatmap (Fig. 3A, left panel) and was tested for group difference. Eleven modules showed statistically significant in CNM-CWM (FDR ≤ 0.05, Student's t-test) and 2 modules in CLM-CWM comparison (FDR ≤ 0.05, Student's paired t-test). Only one module, GM6, showed the significant difference in both CNM-CWM and CLM-CWM comparison (Supplementary Table S4). The relationship of each module with phenotype—“Metastasis” was measured as correlation coefficient (Fig. 3A). Seven modules have positive correlation and 6 modules have negative correlation with “Metastasis” (*p* ≤ 0.01). Hierarchical clustering of modules and Metastasis status showed two large branches (Fig. 3A, right panel). Branch 1 consisted of “Metastasis”—and modules GM1 to GM6, GM9 and GM12. We noticed that 6 out of 8 modules in Branch 1 were positively correlated with metastasis status and were upregulated in either CWM or CLM, or both groups. Branch 2 comprised of the remaining modules, and most were negatively correlated with metastatic status and were downregulated in either CWM or CLM, or both. Positive correlation and clustering with “Metastasis” status in Branch 1 are suggestive of these modules having positive effects on “Metastasis.”

Enrichment analysis identified over-represented Gene Ontology (GO) terms for each module. The most significant GO biological processes or molecular functions, which were identified to positively correlated with metastasis were extracellular matrix organization, angiogenesis, and growth factor binding (GM1), inflammatory response/immune response (GM2), lymphocyte activation/adaptive immune response (GM3), and digestive system development/fibroblast proliferation (GM6). The most significant GO terms identified on negatively correlated modules were response to type I interferon (GM4), ribosome (GM7), and cell division and DNA replication (GM16) (Supplementary Table S4 & S5).

In order to characterize the cell-type specificity of each gene module, we used available data from FACS-purified cells generated from 14 primary CRC samples to compare the module enrichment in various cell subpopulations^9^ (data from GSE39396). As shown in Fig. 3B, three modules, GM18, GM22 and GM24, were highly enriched in epithelial tumor cells. Six modules, GM1, GM2, GM3, GM4, GM6, and GM13 showed strong TME—stromal cell enrichment that including endothelial cells, CAFs and leukocytes (FDR ≤ 0.05; one-way ANOVA test; Supplementary Table S6). The similar cell-type enrichment patterns for most of 26 identified modules were observed in a single-cell CRC RNA seq data^22^ (data from GSE81861), which further validated our observation (Fig. 3B, bottom panel). To further understand the immune cell related modules, we deconvoluted gene modules that showed leukocyte-specific modules using purified immune cell subsets^23^ (data from GSE3982). This included cells from both myeloid lineage such as dendritic cells (DCs), eosinophils, mast cells, macrophages, natural killer cells (NK), and neutrophils and lymphoid lineage such as B cells, NK cells, T helper 1 (Th1), T helper 2 (Th2), T central memory (Tcm), and T effector memory (Tem) cells. We observed that genes of module GM2 were highly expressed in the myeloid phagocytic cells including macrophages and dendritic cells (Fig. 3C), GM3 was enriched in lymphoid NK cells, T cells, and B cells, and GM5 was enriched for neutrophils and eosinophils. In contrast to GM2, the genes of GM4 were highly expressed in three types of immune cells (macrophages, dendritic cells, and neutrophils), which is suggestive of GM4 being a pro-inflammatory module.

We also overlapped TME-related modules with well-defined signatures for angiogenesis^24^, EMT^25^, immune-phenotypes^26^ and T cell exhaustion^27^ to further refine the module functional annotation^28^ (overlap = number of signature genes/ number of genes in module). GM1 overlapped with EMT (*p* < 0.001) and angiogenesis (*p* < 0.001). GM2 overlapped with M0, M1 and M2 macrophages (*p* < 0.001), resting or activated dendritic cells (*p* < 0.001), and neutrophils (*p* < 0.001). GM3 overlapped with different types of T cells and NK cells (*p* < 0.001), B memory cells and M1 macrophages (*p* < 0.001) (Fig. 3D). The later had predominant functional profile that was related to infiltrating B cells and cytotoxic (CD8+) T cells (Supplementary Table S4).

### Transcriptional networks decipher the ecosystem of CRC metastasis

Cancer cell invasion and metastasis are regulated by tumor ecosystem^29^. The TME-enriched gene modules identified in this study give us a unique opportunity to examine the metastatic tumor ecosystem.

**Figure 4:**
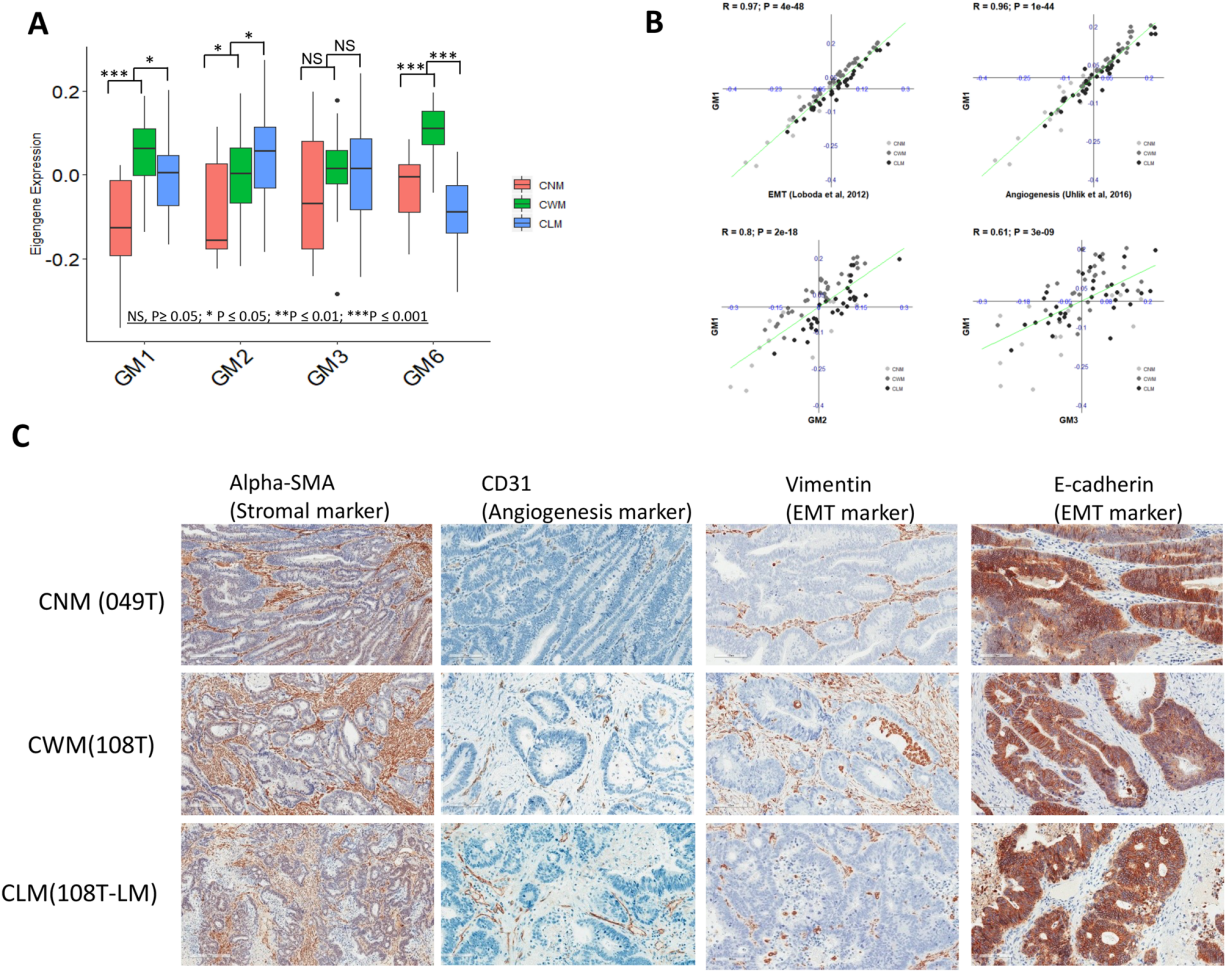
The expression changes of TME—enriched modules in CNM, CWM and CLM. (**A**) Boxplots show the relative expression levels of GM1-3 and GM6. The p value is generated from group comparison. (**B**) Scatterplots show that GM1 is highly correlated with recently published angiogenesis signature^24^ and EMT signature^25^. GM1 also has a strong correlation with immunity-related module GM2 and GM3. (**C**) Representative IHC staining of stroma, angiogenesis and EMT markers in tumors of CNM, CWM and CLM.

*GM1 presents the molecular programs that facilitate cell migration and invasion*: Functional annotation indicated the largest module GM1 was associated with multiple biological processes, including EMT, angiogenesis, ECM remodeling and growth factor-releasing mechanism (Supplementary Table S4 & S7; Supplementary Figure S3A). As shown in Fig. 4A, GM1 was up-regulated in CWM and then slightly down-regulated in CLM. Correlation analysis showed that the expression of GM1 was significantly correlated with the signature of EMT (R = 0.96, *p* = 1e−44; Fig. 4B) and angiogenesis (R = 0.97, *p* = 2e−46; Fig. 4B). IHC staining of a neovascularization marker (CD31) and EMT markers (E-cadherin and Vimentin) in tumor tissues (Fig. 4C) provided the similar expression trajectories of EMT and angiogenesis presented by GM1.

*GM2 and GM3 institute innate and adaptive metastatic immune environment, respectively*: We showed that GM2 was highly expressed in the phagocytic cells (macrophages, neutrophils, and dendritic cells) and GM3 was mainly enriched in T cell population. As shown in Fig. 4A, the GM2 genes were upregulated in CWM and consistently extended to CLM, (Fig. 4A). By examining the gene composition of GM2 and GM3, we observed enrichment of known immunosuppressive genes in modules GM2 and GM3^26,30^ (Supplementary Table S4 & S7; Supplementary Figure S3B & 3C). For example, some immune inhibitory genes, LILRB1-4, LILRA2, SIRPB1, TLR1-2, TLR4-8, VSIG4, TSC22D3, PDCD1LG2, HAVCR2, and LAIR1 were included in GM2, whereas GIMAP4, GIMAP6-8, IL10RA, LAG3, KLRB1, IL2RA, IL2RB, CTLA4, and PDCD1 were bonded in GM3. Although GM3 genes as a group showed no statistically significant difference, many of the important genes mentioned above were enriched in CWM. We also observed that GM1, GM2 and GM3 were highly correlated to each other (Fig. 4B), suggesting these modules may constitute a biological program of invasiveness in the metastatic microenvironment.

*GM6 is strongly associated with the role of CAF*: We found that GM6 was the most correlated and closely clustered module with metastasis in our analysis. Cell type deconvolution showed that GM6 was predominantly enriched in the FACS-purified CAFs and the fibroblasts in the single cell RNA sequencing of primary CRC (Fig. 3A, B). The expression of GM6 genes was significantly high in CWM (Fig. 4A). Stromal marker (alpha-SMA) IHC staining also showed that CWM had the highest stroma content compared to other two groups (Fig. 4C). GM6 contained FGFR1, MMP2, FGF7, FOXF1, PDPN, and WNT5A (Supplementary Table S4 & S7; Supplementary Figure S3F). These six genes were previously found to express in CAFs and promote metastasis in different types of tumors^31–33^.

**Figure 5:**
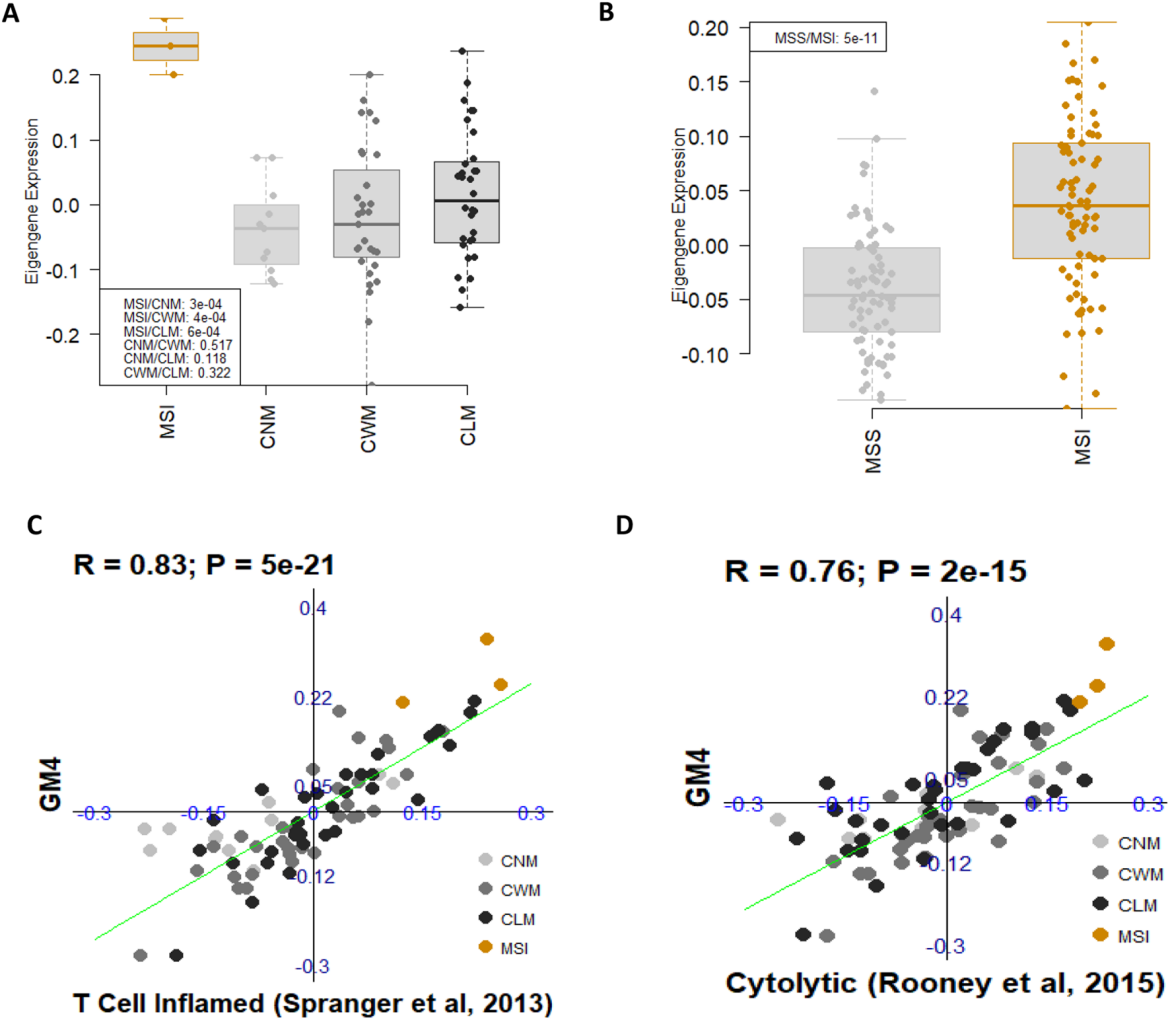
MSI-enriched immunity and module GM4. (**A** & **B**) GM4 is highly expressed in three MSI samples (**A**) and validated in an independent dataset (**B**) data from GSE13294). The p value is generated from group comparison (Welch’s t-test) and is listed in the legend box. (**C** & **D**) Scatterplots show that GM4 is highly correlated with T cell inflamed signature (**C**) and Cytolytic score signature (**D**).

*GM4 recapitulates the immune microenvironment of MSI*: The microsatellite instability (MSI) subset of CRC exhibits an active Th1/CTL immune microenvironment, likely due to the recognition of a high number of tumor-neoantigens^34^. We notified that GM4, a module highly expressed in CD45+ leukocyte (Fig. 3B), was significantly up regulated in 3 MSI samples compared to the rest of samples (Fig. 5A). Since we only had 3 MSI samples in this study, we re-examined the expression of GM4 in an independent dataset that had 78 MSI samples^35^ (data from GSE13294). We found that GM4 was indeed highly expressed in MSI samples (Fig. 5B). The data suggested that module GM4 captured the biological specifics for the immune microenvironment of MSI CRC subset. Further functional enrichment analysis suggested that the major biological function of module GM4 was type I interferon signaling pathway (Supplementary Table S5). Going through the gene composition of this module, we identified several interferon signaling pathway members and immune genes such as CD274 (PD-L1), B2M, IDO1, STAT1, JAK1, BTN3A1-3 and HLA class (Supplementary Table S4 & S7; Supplementary Figure S3D). As shown in Fig. 5C, D, GM4 was strongly correlated with “hot” tumor T cell inflamed signature^36^ (R = 0.83, *p* = 5e−21) and local immune cytolytic Activity^37^ (R = 0.74 and *p* = 7e−15).

### The TME-enriched modules were strongly associated with tumor progression and clinical outcome

**Figure 6:**
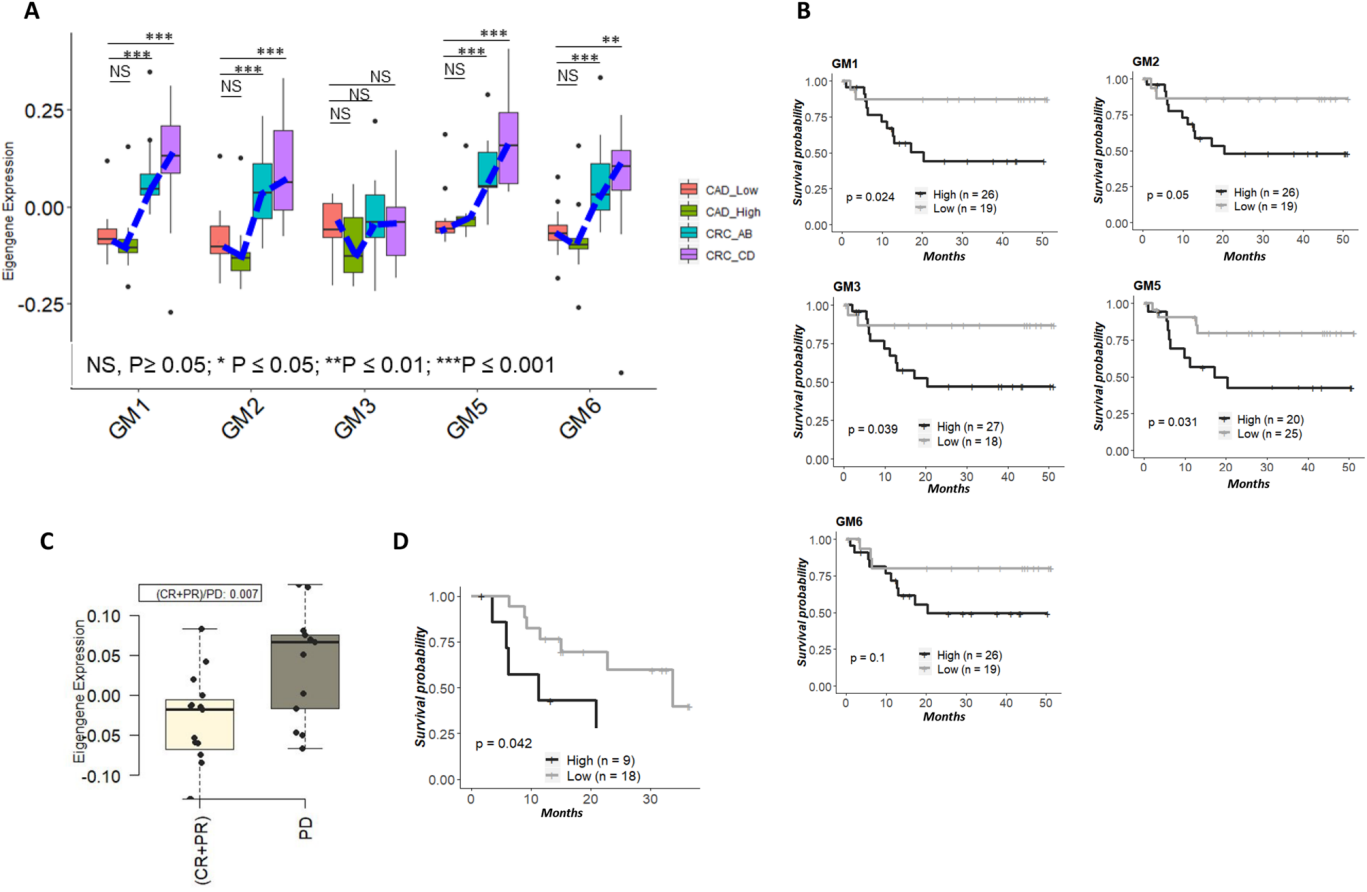
The association of TME—enriched module expression, tumor progression and patient outcome. (**A**) Boxplots show the relative expression of TME-enriched modules in 27 of adenomas with low or high grade (labeled as “CAD_Low” or “CAD_High”) and 25 of carcinoma samples with Duke stage AB or CD (labeled as “CRC_AB” or “CRC_CD”) (data from GSE37364). The expression trajectory of each module is indicated by a dot blue line. The p value is generated from group comparison (Welch’s t-test). (**B**) Kaplan–Meier analysis comparing survival of patients having either high or low TME-related module scores. The dichotomized indicator representing “High” and “Low” module expression was created by “mean” split on module summarized scores. Patients with high scores of GM1, GM2, GM3, and GM5 were associated with poor disease-free survival (PFS). (**C**) Patients (data from GSE78220) with high GM1 expression in melanoma have poor anti-PD-1-treated response (boxplot) and poor overall survival (Kaplan–Meier curve). Log-rank test and Kaplan–Meier survival curves were plotted using R package survival (version 2.44.1.1; https://cran.r-project.org/web/packages/survival/index.html ).

We next examined whether these TME-enriched and metastasis-positively correlated modules (GM1, GM2, GM3, GM5, and GM6) were progressively up or down regulated throughout tumor progression. We plotted the eigengene values of these specific modules across different stages of patients with adenoma or colorectal cancer^38^ (data from GSE37364). As shown in Fig. 6A, the TME-enriched modules except GM3 were significantly down-regulated in adenomas and then were progressively upregulated in carcinomas (*p* ≤ 0.05). This is suggestive of progressive upregulation of TME-enriched modules recapitulating the molecular processes that underlie the transformation of colon tissue from benign adenoma to malignant carcinoma.

The close association of TME-enriched modules and tumor metastasis motivated us to evaluate the prognostic relevance of these modules using Kaplan–Meier analysis with clinical outcome (Supplementary Table S1). For most of the CRC patients involved in this study, we collected long term survival data. We performed “mean” split on module eigengene to create a dichotomized indicator representing “High” and “Low” module expression groups. As shown in Fig. 6B in 45 patients with available follow-up survival data, patients with high scores of GM1, GM2, GM3, and GM5 were significantly associated with poor survival (log-rank test; the p value was 0.024, 0.05, 0.039, and 0.031, respectively). Similarly, the high score of GM6 in these patients trended towards poor survival (*p* = 0.1).

Immune checkpoint blockade resulted in durable antitumor activity in many advanced malignancies. However, efficacy of these agents in solid tumors including MSS CRC has been limited. We further analyzed published clinical data^39^ (GSE78220) and revealed that GM1 was highly expressed among non-responding patients relative to responding pretreatment patients (Fig. 6C), and melanoma patients with high GM1 expression also had poor overall survival to anti-PD-1 therapy (Fig. 6D). Our analysis suggested that GM1 might be associated with resistance to current PD-1/PD-L1 immune checkpoint blockade therapy.

### Construction of a global transcriptional network has identified molecular links of metastasis-associated modules

**Figure 7:**
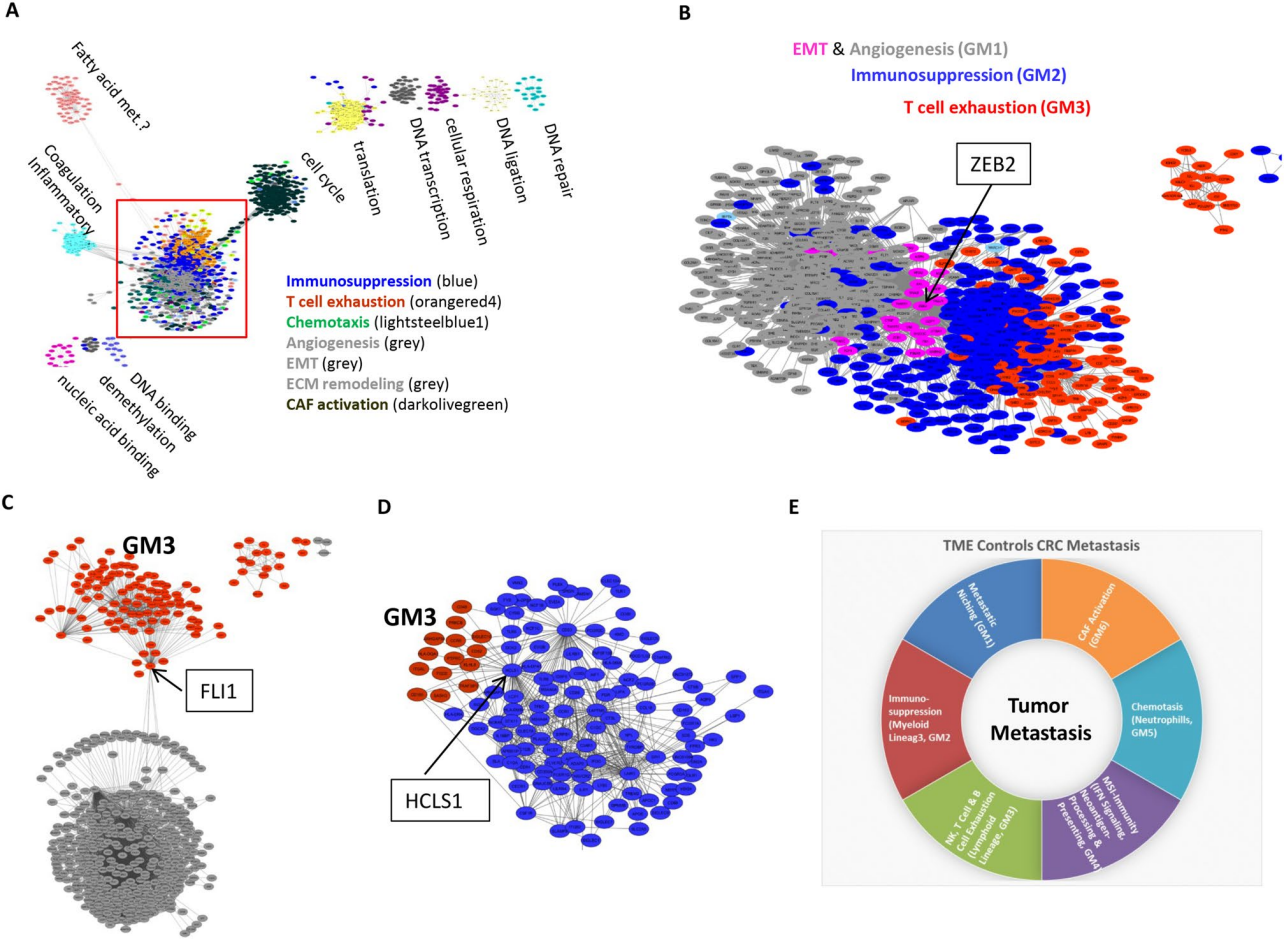
Global co-expression network architecture in CRC metastasis. (**A**) Node color corresponds to each module identified. The size of each subnetwork reflects the number of genes in each module. This network with one larger component, connected to one mid-sized component, and several small islands. The mid-sized component mostly consists of cell cycle genes. Several small islands positioned next to each other for DNA repair, ligation, translation and transcription. The TME—enriched modules such as EMT and angiogenesis, innate immunosuppression, T cell exhaustion are intertwined and interacted together to form the largest component in this global network. (**B**–**D**) Interconnectivity analyses on transcriptional factors that regulate EMT, angiogenesis, immunosuppression, and T and B exhaustion: ZEB2 is highly connected immunosuppression module GM2 to EMT and angiogenesis module GM2. Genes plotted in brown, blue, grey and magenta are unique to the top neighbors of the T cell and B cell exhaustion (GM3), innate immunosuppression (GM2), angiogenesis and EMT (GM1) networks, respectively; ZEB2 is at the center of plots (**B**). FLI1 bridges GM1 to GM3 (**C**). HCLS1 links GM2 to GM3 (**D**). All network graphs (**A–D**) were produced using Cytoscape^56^ (version 3.1.1.). (**E**) A chart to illustrate the hypothetical driving forces of metastasis in CRC TME.

We further constructed a global transcriptional network to illustrate the biological connections among these gene modules. As shown in Fig. 7A, this network had one large component complex connected to one mid-sized component complex and several clusters. The mid-sized component complex mostly consisted of cell cycle genes enriched in module GM16. Several clusters in this network reflected shared functionality due to distinct, but related processes—such as DNA damage repair, ligation, translation, and transcription. Importantly, the TME-enriched modules such as EMT and angiogenesis (GM1), innate (GM2) and adaptive immunity (GM3), CAF activation (GM6), MSI-enriched type I IFN signaling (GM4), and chemotaxis (GM5) were all intertwined together to form the largest component complex in this global network (Fig. 7A, B). This global transcriptional network revealed putative functional interactions and gene modules associated with metastatic process, and the size of each complex in the network indicated its importance in tumor metastasis.

To further identify interactive relationship and the potential key regulators among cancer gene modules, we examined the intra–modular connections among GM1, GM2, and GM3. Interestingly, we noticed that ZEB2, a known transcriptional factor associated with EMT, was a key hub gene that connected these modules^40^ (Fig. 7B). ZEB2 has recently been shown to be required for terminal differentiation of T cells^41,42^ and the maturation of NK cells^43^. ZEB2 has also been identified in transcriptional analyses as a potential transcriptional factor involved in dendritic cell^44^ and macrophage development^45^. Additionally, we also observed that a hub gene, FLI1 that connected module GM1 with GM3 (Fig. 7C). FLI1 is a member of the ETS transcription factor family, and ETS factors are essential for maintaining vascular homeostasis and immune system regulation. Of the 29 recognized ETS factors, nine are known to regulate genes involved in immunity^46^. Our network analysis indicated a key regulatory role of FLI1 played in the connection of angiogenesis and T cell function. Additionally, we found that Hematopoietic lineage cell-specific protein 1 (HCLS1), an actin regulatory protein, also was a hub gene that bridges GM2 and GM3 (Fig. 7D).

To illustrate the potential driving forces of metastasis in CRC, we hypothesized and constructed a diagram to illustrate the contribution of six gene modules in CRC metastasis (Fig. 7E). These categories were abstracted mainly based on the expression and functional identities of the representative genes, related cell-type specificities, and gene ontology of TME related modules.

## Discussion

The molecular mechanisms of cancer metastasis remain elusive although numerous efforts have been attempted to address them. In this study, we have identified that TME-enriched modules are positively correlated with metastasis and are highly expressed in metastatic groups (CWM and/or CLM). The results imply that the tumor microenvironment components, such as immune-suppression, EMT and angiogenesis, are associated with dissemination and distant metastasis of CRC.

By systematically and unbiasedly analyzing the exome and RNA sequencing and SNP6.0 data, we assessed any potential molecular mechanisms that might associate with CRC metastasis. First, we observed a high degree of similarity in genomic alterations between CWM and CNM or between paired CWM and CLM, although considerable genetic heterogeneity was observed within the group. Typically, mutations found in metastases were also present in the matched primary tumors. Second, we compared the transcriptome profiles among the groups and found that EMT was the most significant difference between CWM and CNM. Applied co-expression network analysis, we found that the molecular modules associated closely with metastasis were majorly TME-related. We showed that GM1, a gene module associated with many TME events, and GM6, a gene module associated with CAFs, were enriched in metastatic tumors. Deconvolution of leukocyte-enriched modules with gene signatures of purified immune cells, we identified that GM2 was enriched for myeloid cells, GM3 for lymphoid cells, and GM5 for neutrophil. We also revealed that GM1, GM2, GM3 and GM6 were closely correlated with CRC metastasis. Our approach identified a set of transcriptional networks and biomarkers that were specific for a certain cell types from a mixture of tumor and stromal tissues, which overcame the major hurdle in the analysis of tumor gene expression data. It may shed some light on understanding the role of each cell type and its contribution to the metastatic process. Third, we asked whether any key gene critical for tumor metastasis can be identified from the global transcriptional network. The current study provided a set of insights from our identification of hub genes central to each network, including transcription factors, which would not have been identified using conventional approaches. For example, our approach identified ZEB2 as a key hub gene linked to an integrated network that orchestrates the molecular processing of metastatic cascade.

Interestingly, we revealed that GM4 was significantly upregulated in our MSI CRC samples and an independent CRC cohort^35^. MSI resulted from defects in the DNA mismatch repair system^47^. Growing evidence showed that MSI colorectal carcinomas were associated with high-level immune infiltrates^48^. Our study found that GM4 enriched patients were tightly correlated with inflamed or local immune cytolytic phenotype tumors, consistent with previous observations^30^. The immune-inflamed phenotype was characterized by the presence of both CD4 and CD8 expressing T cells in the tumor parenchyma, and these immune cells were positioned in proximity to the tumor cells^36^. This profile suggests the presence of a pre-existing anti-tumor immune response in MSI CRC cancers. Indeed, clinical responses to anti-PD-L1/PD-1 therapies occurred most often in patients with MSI inflamed tumors^48^.

We showed that GM2, a myeloid cells gene module, and GM3, a T cell gene module, were positively correlated with tumor metastasis. Some immune inhibition markers (LILRB1-4, LILRA2, SIRPB1, TLR1-2, TLR4-8, LAPTM5, VSIG4, TSC22D3, PDCD1LG2, HAVCR2, and LAIR1) in GM2 were up-regulated in metastatic group CWM / CLM, which evidenced the existence of inhibitory effects on antigen-presenting cell phenotype and subsequent T-cell responses in metastatic TME^49^. We also notified that some GM3 characteristic genes (GIMAP4, GIMAP6-8, IL10RA, LAG3, KLRB1, IL2RA, IL2RB, CTLA4 and PDCD1) were upregulated in CWM, and these genes were associated with T cell and B cell negative regulation and immune exhaustion^49^.

Our transcriptional comparison demonstrated that EMT and angiogenesis were the most significant pathway differences between CWM versus CNM and CLM versus CWM, respectively. Accumulated reports suggest that EMT is a key process in which cancer cells transit into highly invasive cells for dissemination, while the accompanied angiogenesis is important for tumor development, as tumors must establish a blood supply for growth^14^. Although tumor cells are believed to engage in tumor angiogenesis, studies have shown that the tumor microenvironment and infiltrating immune cells are also important for regulating tumor angiogenesis. The infiltrating immune cells are crucial for regulating the formation and the remodeling of blood vessels in the tumor^50^. GM1 defined in this study captures the overall dynamic transcriptional programs and the reciprocal interactions of tumor cells with ECM, CAF, mesenchymal tumor cells, endothelial cells, and tumor-associated macrophages.

We revealed that GM6 was strongly correlated with metastasis and highly enriched in CAF. It is becoming increasingly clear that CAF is one of the crucial components in TME. It promotes tumor growth through stimulation of tumor cell proliferation, enhanced angiogenesis, and ECM remodeling^51^. Moreover, CAFs mediate tumor-promoting inflammation and modulate the components of the inflammatory microenvironment that facilitates tumor initiation, progression, and metastasis^52^.

In conclusion, we have identified TME gene modules of EMT, angiogenesis, CAFs, and immune suppression, as the key events closely associated with CRC metastasis, suggesting that tumor metastasis is a complex process engaging tumor cells, immune cells, endothelial cells and their interactions in tumor microenvironment. It is necessary to explore a strategy of combining targeted therapy, immunotherapy, and anti-angiogenic therapy for effective treatment of metastatic CRC.

## Materials and methods

### Sample preparation

Thirty-two matched liver metastases, CRC primary tumors, and normal tissues were collected at Samsung Medical Center (SMC). Thirteen CRC primary tumors without distant metastasis and matched normal tissues were also collected as a control at SMC. Licensed pathologists confirmed the histologic diagnoses and estimated all the formalin-fixed paraffin-embedded samples with purity of ≥ 40% according to H&E staining. Written informed consent was obtained from all participants. All methods were carried out in accordance with relevant guidelines and regulations, and all experimental protocols done in the study were approved by Samsung Medical Center. The whole-exome and RNA Sequencing, and SNP6.0 analysis were conducted for all samples (Supplementary Figure S1).

### Immunohistochemistry

Immunohistochemistry (IHC) was performed on 4 μm sections of formalin-fixed, paraffin-embedded tissue. Bond-max autoimmunostainer (Leica Biosystem, Melbourne, Australia) with Bond Polymer refine detection (DS9800, Vision Biosystems, Melbourne, Australia) and Ventana BenchMark XT automated slide processing system (Ventana Medical Systems) were used according to the manufacturer’s protocol. The primary antibodies were mouse monoclonal antibodies for alpha-SMA (DAKO, 1:1,000 dilution), E-cadherin (4A2, Cellsignaling, 1:200 dilution), CD31 (DAKO, 1:200 dilution), Vimentin (DAKO, 1:1,000 dilution) and CD8 (SP57) rabbit monoclonal antibody (Ventana, 1:200 dilution). The results were evaluated by pathologist without prior knowledge of the clinicopathological or molecular data.

### Whole-exome sequencing

Genomic DNA for all samples was hybridized using Agilent SureSelect Human All Exon v4 (51 Mb) kit. The enriched DNA fragments were sheared to 150-200 bp and subjected to standard Illumina Genome Analyzer library preparation according to Illumina's protocol. Sequencing depth of 120X for tumor and 80X for normal for the whole-exome was generated on Illumina Hiseq 2000 platform.

### Somatic mutation analysis

Genomics reads were aligned to the human reference genome (hg19) with BWA-MEM (https://bio-bwa.sourceforge.net/). Somatic single nucleotide variant (SNV) was detected by VarScan2 (https://varscan.sourceforge.net/). The preliminary parameters were set as (1) minimum supporting reads in tumor ≥ 8; (2) minimum supporting reads in normal ≥ 6; (3) minimum allele frequency in tumor ≥ 0.1; (4) maximum allele frequency allowed in normal ≤ 0.1; 5) *p* value ≤ 0.05. To further reduce the false positive of SNV sites, we filtered SNV sites by more stringent criteria: (1) site with Fisher’s exact test *p* value ≤ 0.05; (2) minimal distance between the SNV-base and the read end (or beginning) ≥ 5; (3) site should pass 3 more statistical tests for base quality, mapping quality, and strand bias.

To identify somatic indels, gap allowed alignment was performed using Burrows-Wheeler Aligner (BWA) as described previously; indels were then identified using the GATK package (https://www.broadinstitute.org/gatk/) in a somatic mode based on the local realignment results. The windows size is set by 300 bp.

### Mutation annotation

ANNOVAR (https://www.openbioinformatics.org/annovar/) was used to annotate all mutations. The mutations deposited in COSMIC (v64 release) and dbSNP (v135 release) database were marked by their mutation ID. We utilized the method of Youn and Simon^53^ to predict the significance of gene mutations, and a mutation score was calculated based on BLOSUM80 in the following order: missense < inframe indel < mutation in splice sites < frame shift indel = non-sense.

### Copy number analysis

Patient’s DNA were run on Affimetrix’s Genome-Wide Human SNP 6.0 microarray, according to the protocols recommended by its manufacturer (https://www.affymetrix.com/support/technical/byproduct.affx?product=genomewidesnp_6). Then, the raw SNP6 CEL image files generated from the above SNP6 run were used as the input to run GenePattern’s Affymetrix SNP6 Copy Number Inference Pipeline (https://www.genepattern.org/affymetrix-snp6-copy-number-inference-pipeline). This pipeline generated segmented copy number regions across the whole genome for each sample. Entrez gene models were used to assign copy number to genes located on a segmented copy number region. For this study, gene’s copy numbers were classified into 5 categories: deletion (CN < 1.0), loss (1.0 ≤ CN < 1.85), diploid (1.85 ≤ CN ≤ 2.15), gain (2.15 < CN ≤ 4.0), and amplification (CN > 4.0). When a gene contains more than one copy number region, the number which is most deviate from 2 is selected. However, if a gene contains a segment of CN < 1.0, such gene is classified as Deletion.

### RNA sequencing

RNA-Seq was performed on an Illumina HiSeq. 2000 with the Illumina TruSeq RNA Sample Preparation Kit v2 as described previously^54^. Paired-end sequencing with a read length of 100 bp and targeted read depth of 50 million reads/sample was performed. Data were filtered to remove genes with fewer than 5 counts across 80% of the samples from the analysis. The resulting data were quantile-normalized and summarized across samples. Co-expression network analysis was carried out using genes with relatively high signals (15,208 genes, signals > 5 units across 80% of the samples).

All differentially expressed gene analysis was conducted using the DESeq2 package^20^. Fold change (FC) from comparisons were calculated to show up- or down-regulation of genes between CLM and CWM or CWM and CNM. Raw p-values were adjusted separately for each comparison using the False Discovery Rate (FDR) with Benjamini–Hochberg testing correction. Differentially expressed genes (DEGs) were identified from comparisons when FDR < 0.05 and |FC|≥ 2.

### Analysis of transcriptional gene networks by weighted co-expression network construction method

Transcriptional co-expression networks were constructed using the weighted gene co-expression network analysis (WGCNA) Bioconductor method as described previously^55^. To minimize the bias in our analysis, we constructed this co-expression network in an “unsupervised” manner. We only excluded RNA-Seq low count genes (the lower quartile of the whole transcriptome) and did not filter any genes based on any clinical or pathological features, which resulted in modules' gene composition up to the genome scale (total 15,208 genes). Pearson correlation coefficients were calculated for all possible pairs of genes across all samples. The correlations matrix was raised to a soft threshold power 6 based on the criterion of approximate scale-free topology, thus producing a weighted network^17^ (weighted correlation = correlation^6^). The weighted network was transformed into a network of Topological Overlap (TO)—an advanced co-expression measurement that considered not only the correlation of 2 genes with each other but also the extent of their shared correlations across the weighted network^17^. The modules were then constructed and identified from the resulting topological overlap matrix at several different dissimilarity correlation thresholds, and the threshold of 0.10 was used to merge module boundaries for afterward analysis (Supplementary Figure S2). For each gene, we determined its connectivity within its module of residence by summing up the TOs of the gene with all the other genes in the module. As each module comprises highly correlated genes, their condensed representative expression will be summarized by eigengene profiles^55^ (the red lines in Supplementary Figure S2c & 2d). The eigengene, the first principal component of a given module, may therefore effectively summarize the principle pattern within the cellular transcriptome with minimal loss of information^55^. The summarized module expressions were then correlated with a matrix of clinical variables and the resulting correlation matrix was visualized as a heat map (Supplementary Figure S2b). This dimensionality-reduction approach also facilitated correlation of modular eigengenes with clinical traits. The analysis was performed using R package WGCNA^55^ (version 1.66). All network graphs were produced using Cytoscape^56^ (version 3.1.1.). A full list of genes by module constructed appears in Supplementary Table S7.

### Gene ontology enrichment analysis

We analyzed each module for enrichment in genes with particular Gene Ontology (GO) and compared with the background list of all genes in the whole genome for functional annotation of modules on the basis of their gene composition. Twenty-six modules of genes were identified (Supplementary Table S4). Immunity, angiogenesis, metabolism and cell proliferation were most significant categories among these 26 modules. Although the expression patterns in each module were different, many of the modules shared similar GO categorizations, suggesting that some modules may be functionally related.

### Module analysis

Fisher’s Exact Test used to assess the significance of overlap between modules as described^28^. The expression profiles were summarized by module eigengenes (ME). Pearson’s correlation coefficient between MEs was calculated and used to hierarchically cluster modules^28^. A one-way ANOVA method was used to distinguish the expressed modules among CNM, CWM, and CLM group comparison (Supplementary Table S4). Hub genes were identified and ranked by intra-modular connectivity as defined previously^17^. To calculate the overlap of signatures with modules derived from network analysis, we used the formula described by Mckinney et al^28^. This formula allowed correction for variable module size: [(signature genes overlapping with module genes, n)/(genes in the module, n)] × 100. As a control, the overlap of randomly selected signatures of equivalent size was used and showed adjacent to the above plots.

### Statistical analyses and visualization

Welch’s two-sample t test was used to calculate t-statistics in R^57^. GOstats (R package, Version 3.5.2) was used to identify enriched GO terms in a ranked list by the minimum hypergeometric score. A one-way ANOVA was used to identify module expression among CNM, CWM, and CLM groups. For all applicable statistical tests, a *p* value of 0.05 was the threshold for significance. All heatmaps were generated by using heatmap.2 of gplots (R package, version 3.0.1.1; https://cran.r-project.org/web/packages/gplots/index.html) except Fig. 1A, which was graphed by ComplexHeatmap^58^ (R package, version 1.18.1). The volcano plots in Fig. 2A & 2B were generated using ggplot2 (R package, version 3.2.1; https://cran.r-project.org/web/packages/ggplot2/index.html ). The copy number alteration in Fig. 1C was visualized using Copynumber^59^ (R package, version 1.24.0).

## Supplementary information

Supplementary Tables

Supplementary Figures
