# Supplementary material for: Molecular dissection of CRC primary tumors and their matched liver metastases reveals critical role of immune microenvironment, EMT and angiogenesis in cancer metastasis
Source: Sci Rep. 2020 Jul 1;10:10725. doi: 10.1038/s41598-020-67842-5 (PMC7330040; doi:10.1038/s41598-020-67842-5)
Supplement: Supplementary file 2 — Supplementary Figures [file 41598_2020_67842_MOESM2_ESM.pptx]

## Slide 1
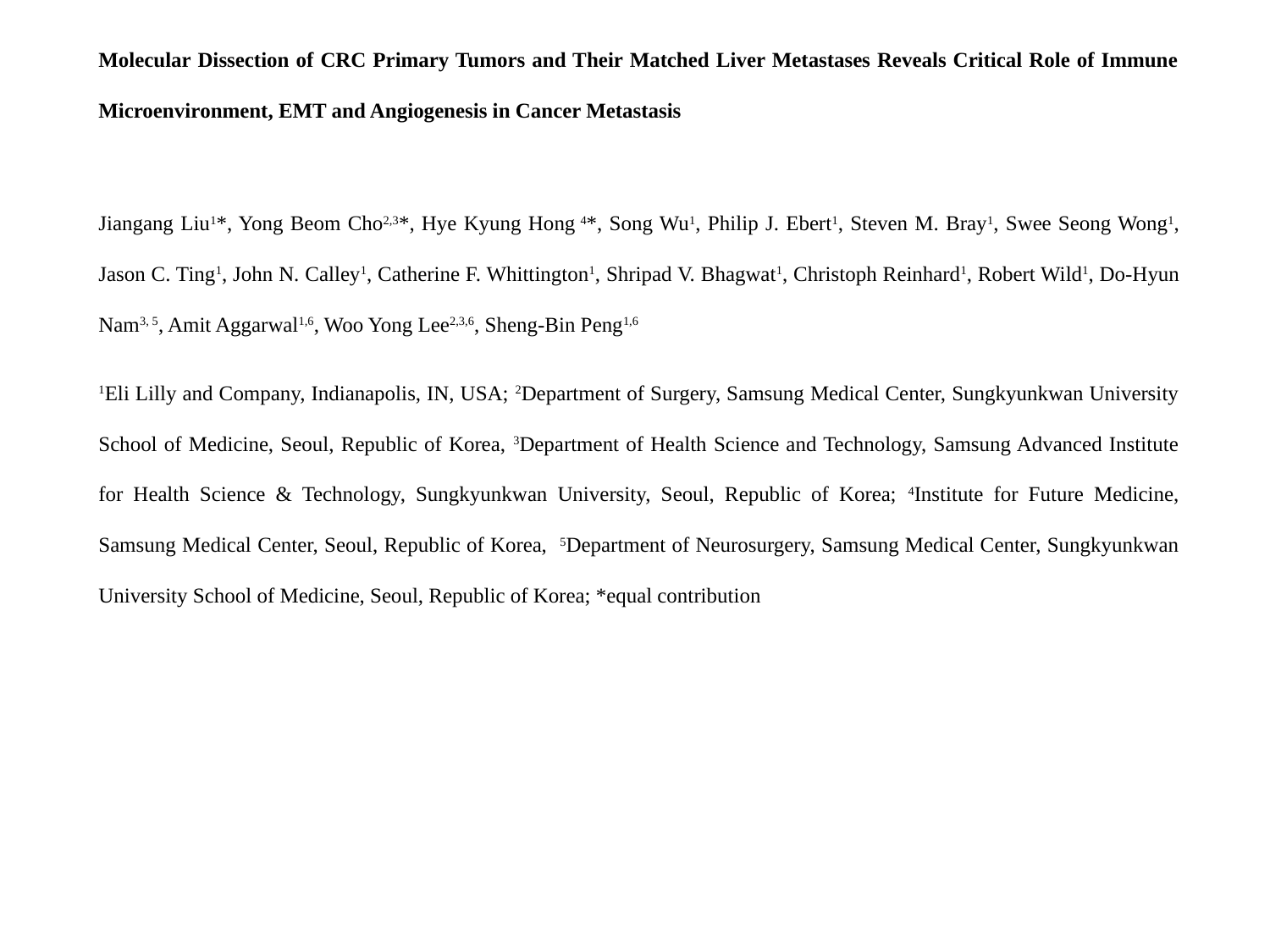

Molecular Dissection of CRC Primary Tumors and Their Matched Liver Metastases Reveals Critical Role of Immune Microenvironment, EMT and Angiogenesis in Cancer Metastasis
Jiangang Liu1*, Yong Beom Cho2,3*, Hye Kyung Hong 4*, Song Wu1, Philip J. Ebert1, Steven M. Bray1, Swee Seong Wong1, Jason C. Ting1, John N. Calley1, Catherine F. Whittington1, Shripad V. Bhagwat1, Christoph Reinhard1, Robert Wild1, Do-Hyun Nam3, 5, Amit Aggarwal1,6, Woo Yong Lee2,3,6, Sheng-Bin Peng1,6
1Eli Lilly and Company, Indianapolis, IN, USA; 2Department of Surgery, Samsung Medical Center, Sungkyunkwan University School of Medicine, Seoul, Republic of Korea, 3Department of Health Science and Technology, Samsung Advanced Institute for Health Science & Technology, Sungkyunkwan University, Seoul, Republic of Korea; 4Institute for Future Medicine, Samsung Medical Center, Seoul, Republic of Korea, 5Department of Neurosurgery, Samsung Medical Center, Sungkyunkwan University School of Medicine, Seoul, Republic of Korea; *equal contribution

## Slide 2
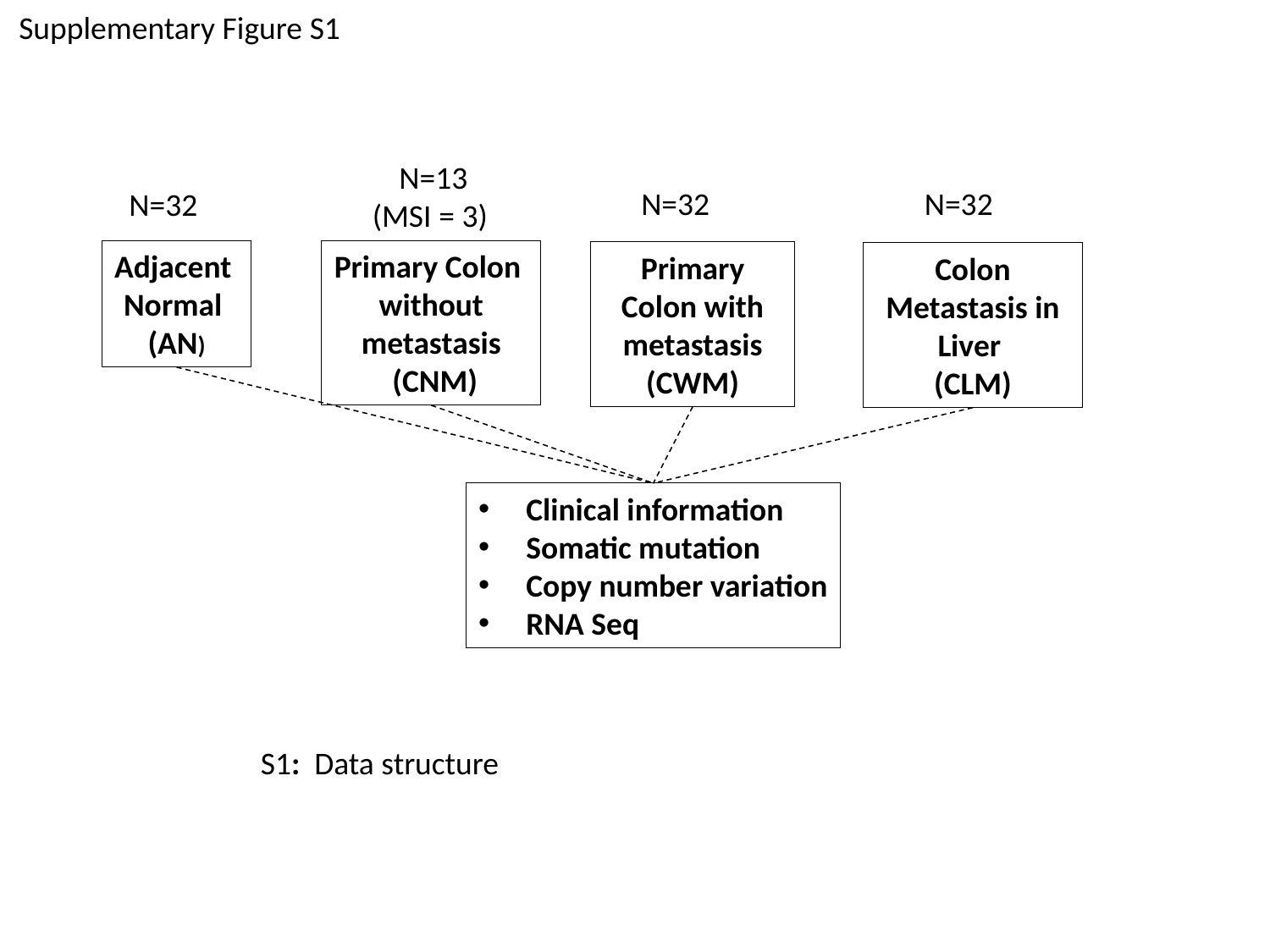

Supplementary Figure S1
N=13
(MSI = 3)
N=32
N=32
N=32
Primary Colon
without metastasis
 (CNM)
Adjacent
Normal
(AN)
Primary Colon with metastasis (CWM)
Colon Metastasis in Liver
(CLM)
Clinical information
Somatic mutation
Copy number variation
RNA Seq
S1:  Data structure

## Slide 3
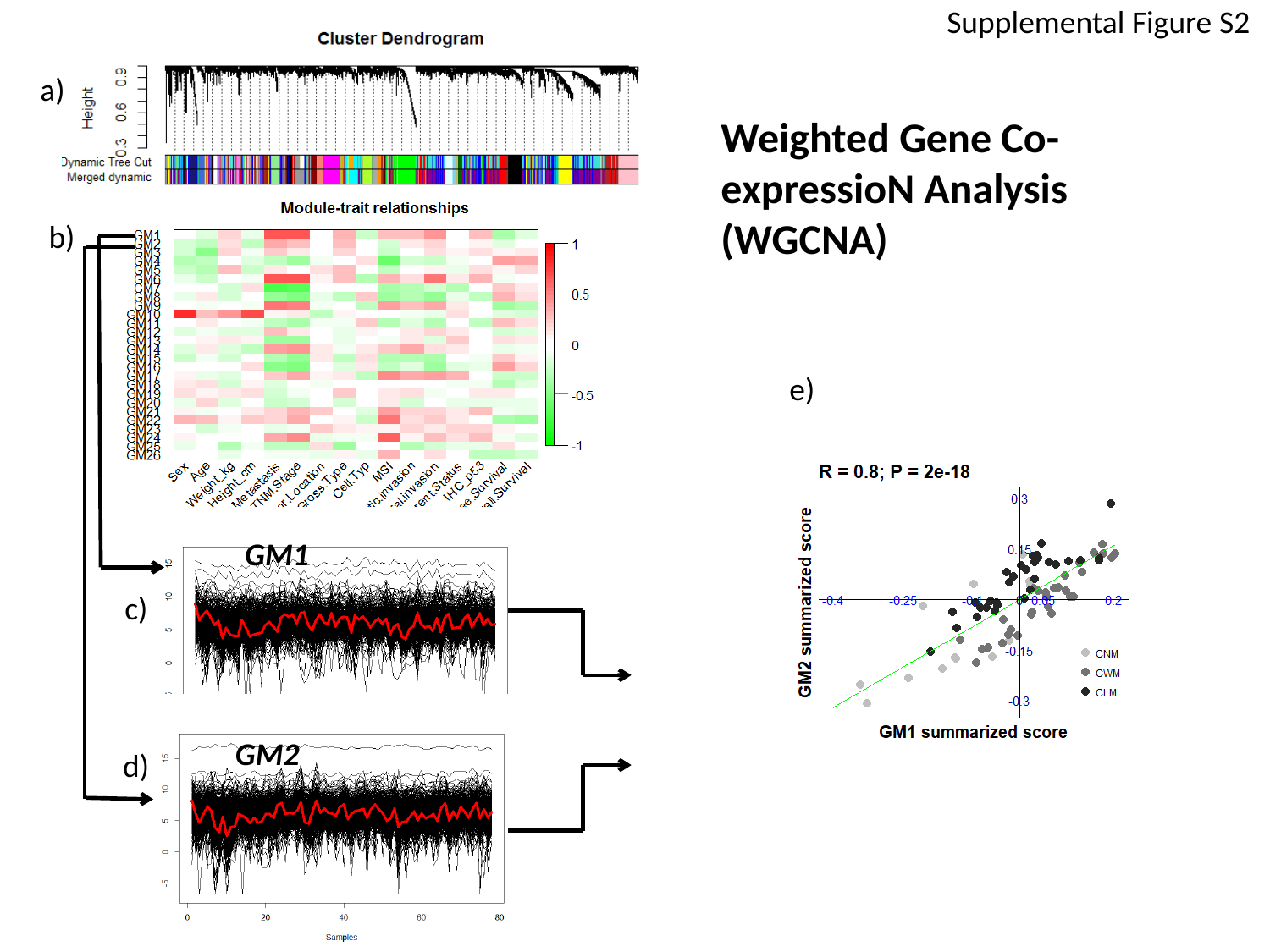

Supplemental Figure S2
a)
Weighted Gene Co-expressioN Analysis (WGCNA)
b)
e)
GM1
c)
GM2
d)

## Slide 4
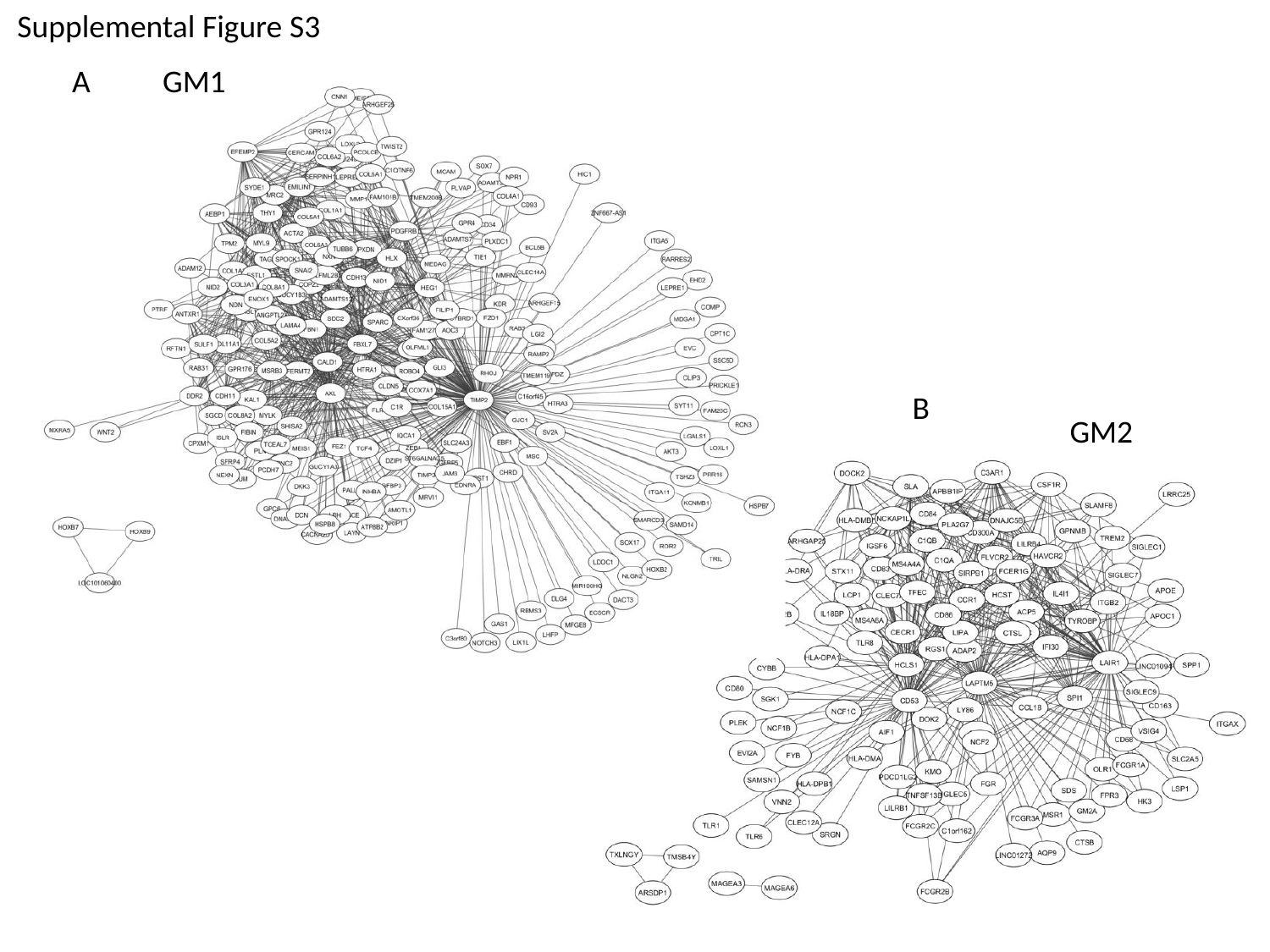

Supplemental Figure S3
A
GM1
B
GM2

## Slide 5
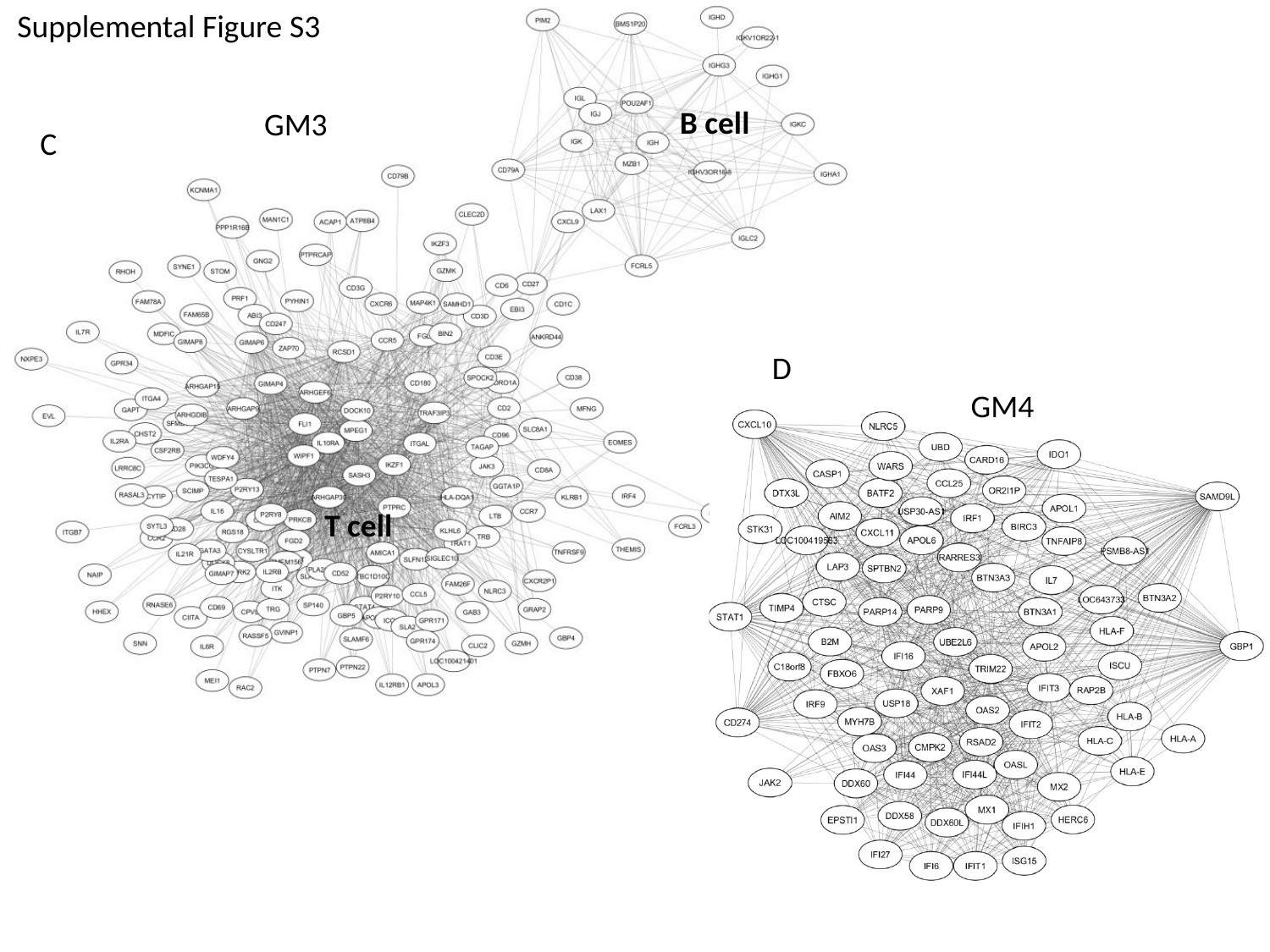

Supplemental Figure S3
B cell
GM3
C
D
GM4
T cell

## Slide 6
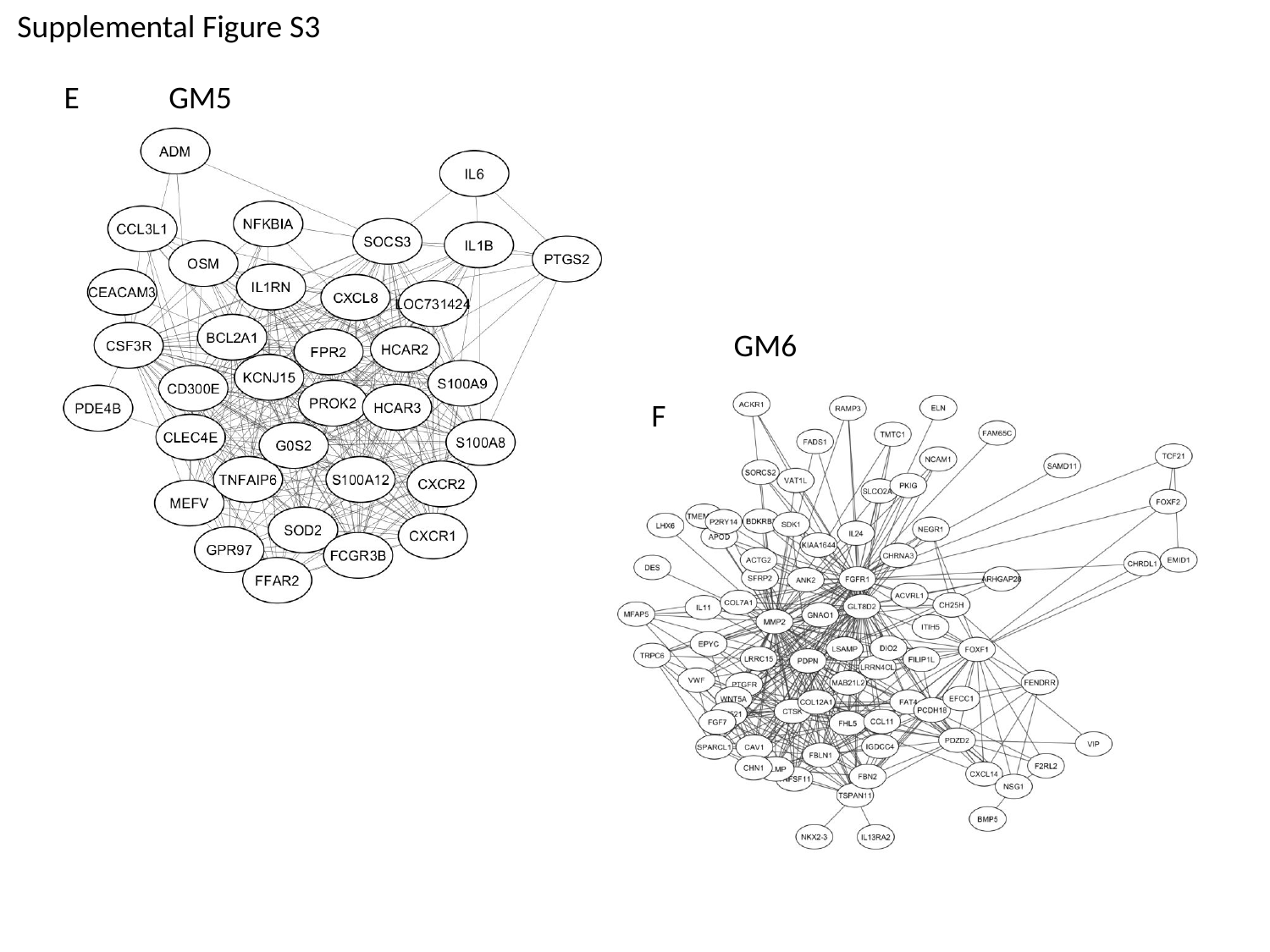

Supplemental Figure S3
E
GM5
GM6
F
